# Electrochemical Bioelectronic Device Consisting of Metalloprotein for Analog Decision Making

**DOI:** 10.1038/srep14501

**Published:** 2015-09-24

**Authors:** Yong-Ho Chung, Taek Lee, Si-Youl Yoo, Junhong Min, Jeong-Woo Choi

**Affiliations:** 1 Department of Chemical and Biomolecular Engineering, Sogang University, Seoul 121-742, Korea; 2 Department of Chemical Engineering, Hoseo University, Asan 336-795, Korea; 3 Interdisciplinary Program of Integrated Biotechnology, Sogang University, Seoul 121-742, Korea; 4 School of Integrative Engineering, Chung-Ang University, Seoul 156-756, Korea

## Abstract

We demonstrate an analog type logical device that combines metalloprotein and organic/inorganic materials and can make an interactive analog decision. Myoglobin is used as a functional biomolecule to generate electrochemical signals, and its original redox signal is controlled with various mercapto-acids by the distance effect between myoglobin and a metal surface in the process of electron transfer. Controlled signals are modulated with the introduction of inorganic materials including nanoparticles and metal ions. By forming a hybrid structure with various constituents of organic/inorganic materials, several functions for signal manipulation were achieved, including enhancement, suppression, and shift. Based on the manipulated signals of biomolecules, a novel logical system for interactive decision-making processes is proposed by selectively combining different signals. Through the arrangement of various output signals, we can define interactive logical results regulated by an inherent tendency (by metalloprotein), personal experience (by organic spacer sets), and environments (by inorganic materials). As a practical application, a group decision process is presented using the proposed logical device. The proposed flexible logic process could facilitate the realization of an artificial intelligence system by mimicking the sophisticated human logic process.

In this era dominated by information and communication technology, digitized arithmetic and logic operations have been intensively developed in the field of silicon-based electronic devices. These developments have contributed to the progress of computing and algorithmic technologies because binary coded signals represented by ‘1’ and ‘0’ can sufficiently define, clarify, and operate on input information by the integration of a single simple unit^1^,^2^,^3^,^4^,^5^. However, a weakness is exposed in silicon-based electronic devices when manipulating non-standardized, continuous, and analog input signals. Particularly, complex processes such as pattern recognition or decision making in the human logic system are difficult to achieve in terms of software and hardware with the current logic systems using silicon-based digital signals. Human logic is not dichotomous and depends not only on individual personal tendencies such as preconceived notions and personality, but also on individual learning experiences or environments. This significant dissimilarity between conventional silicon-based logic tools and natural human logic has become problematic in the development of new human interfacing electronic systems that can be directly connected to human organs. An electronic platform with humanoid logic characteristics is essential to provide analogous logic processes between a human and an electronic device when non-standardized analog tools are used with the transfer, combination, and reinforcement of input signals.

To achieve information processing for an analog electronic device, new applications of biomaterials such as DNA and protein have been demonstrated in electronics, and various concepts using biomaterial have been proposed by applying biomolecules to conventional electronic platforms^6^,^7^,^8^,^9^. Some digital electronic circuits composed of biomaterials have demonstrated the digitalization of analog continuous signals generated by biomaterials by input stimuli (representing information) according to certain classification criteria^10^. For example, electrochemical signals from metalloprotein can be detected as a form of continuous analog signal generated by the reduction and oxidation of metal ions in the structures when a redox potential is applied^11^,^12^. These reactions in metalloprotein are more effective than other physical and chemical reactions because they are already optimized for the processes of transferring energy and mass in natural biological systems.

We demonstrated several limited results using an electrochemical signal from metalloproteins for a bioelectronic device as alternative concepts for memory and processors by converting an analog signal to a digital signal^13^,^14^. However, these simple applications of biomaterials are limited in the application to bio-inspired information processes such as a decision making process that can be affected by personality and environment. Brain-like functions in a chip have recently been conceptually realized by integration with a silicon-based device^15^, but the logical process is not analogous to ambiguous human logic algorithms. Therefore, the main aim of this study is to propose a hardware-type electronic device that is analogous to human logic by combining biological, organic, and inorganic materials.

The proposed logical device uses metalloprotein for an artificial hardware-type interactive algorithm for an analog decision-making process. The main concept is to employ “inherent and experienced human tendencies” and “environments” as well as input information characteristics in a logical bio-device. A human’s final decision output results from a combination of individual personal tendencies and the outer environment, as well as the polarity (positive or negative information), the frequency, and intensity of the input (Fig. 1a left).

We define a human decision result using two different regions: 1) an independent positive or negative region when the same polarity of information input is used (only positive or only negative input), 2) and an interactive region that depends on inherent and experienced tendencies (defined as tendency polarity, TP), environments, and mixed input information (Fig. 1a right). A decision threshold is also defined in each region to provide the decision confidence. Below the threshold is ambiguity, in which a decision trend can be determined but a decision cannot be made. If input information is sufficient to determine a decision (above the threshold), the decision can be made with reliability and confidence, depending on personal tendencies, the environment, and the combination of input information.

To realize this concept, we designed a device consisting of organic spacers, metalloprotein, and inorganic materials. Myoglobin generates redox signals by an applied electrical potential resulting from ionic state change of the metal ion in its structure. It acts as a logical signal generator, which is used to define an inherent human tendency. This redox reaction basically operates on an electrode as a pathway for an electrical field so that signals can be controlled by organic spacer sets through distance control between the myoglobin and a metal surface, which is used to define an experience-induced human tendency. Also, signals controlled by organic materials can be modulated by applying inorganic materials to the biomolecules as an environment-dependent signal modulator. By combining these functions of signal control and modulation, a functional logic unit can be formed using bio-hybrid materials (Fig. 1b left).

Let S1 be a potential-based peak position defined by the result polarity and S2 be a current-based peak intensity defined by the result confidence in the cathodic peak potential (Epc) of myoglobin. These quantities were selected as the output decision result, as shown on the left in Fig. 1c. These two parameters vary according to environmental features, such as organic spacers immobilized on metal surfaces, inorganic materials bound on metalloprotein, and combinations of input information. Multi-electrode patterns (input frequency) have been used for the solid platform, on which metalloprotein has been selectively immobilized based on reductive cleavage (Fig. 1b right). Using the electrochemical signals of myoglobin, we demonstrate device characteristics equivalent to inherent and experienced human tendencies and an environment-dependent interactive decision-making process by signal convergence with overlapping and combining negative and positive recognition (Fig. 1c right).

In this study, we demonstrate that 1) the basic logical functions of output result summation and interaction work with two different input polarities, 2) the interactive region depends on individual personal tendencies, and 3) the interactive region determined by personal tendencies can be changed by environments. Finally, we demonstrate that the proposed device can be directly applied to understand and estimate group decisions by 12 people with different characteristics or environments.

## Results

### Electrochemical signal control and logical output generation

**Figure 1:**
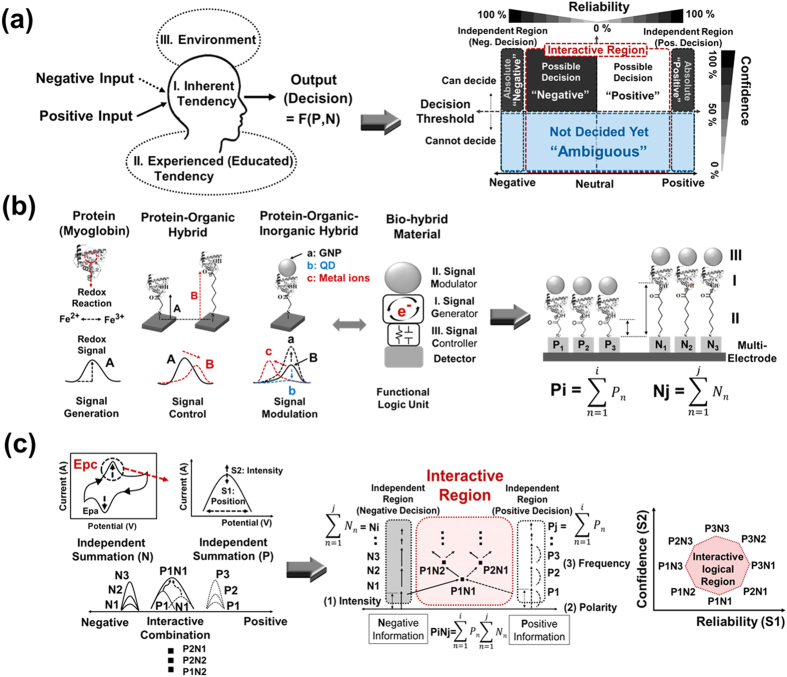
(**a**) Conceptual structure of the human decision-making process (left), defined regions and parameters related to the decision making process (right). (**b**) Formation of bio-hybrid materials for the realization of proposed concept (left), specific device structure as a hardware-type platform for logic operations (right), (**c**) Parameter definition and conceptual functions using output results (left), demonstration of defined regions (independent and interactive) using controlled electrochemical signal of metalloprotein (right).

**Figure 2:**
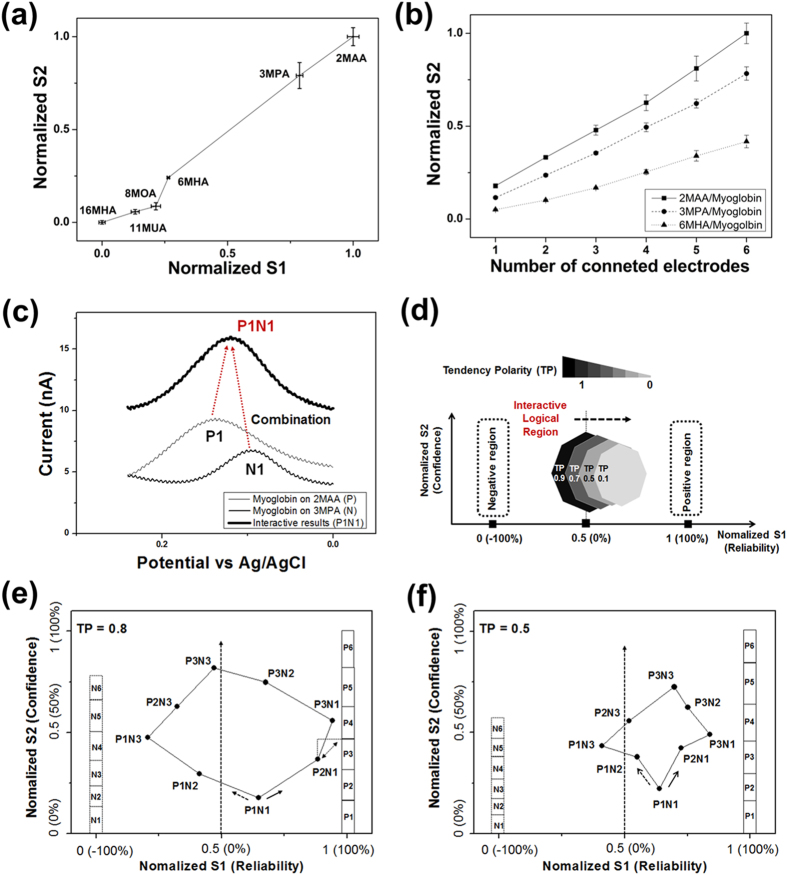
(**a**) Changes of S1 and S1 values of immobilized myoglobin on gold surface due to the length of organic spacers. (**b**) Arithmetic signal increase of myoglobin immobilized on 2MAA, 3MPA, and 6MHA to demonstrate independent regions. (**c**) Combination of redox peaks (P1N1) by merging signals of myoglobin on 2MAA (P) and 3MPA (N). (**d**) Variation of interactive logical regions according to the TP value. (**e**) Balanced device using 2MAA (P) and 3MPA (N) spacer set. (**f**) One-sided device using 3MPA (P) and 6MHA (N) spacer set. Signal variations obtained by combination of positive and negative input signals. The dotted line indicates median value, and the normalized values 1 and 0 represent the perfect positive and negative signals, respectively (P: positive, N: negative). The black arrow and dotted arrow near P1N1 refer to the signal changes in the cases of adding positive and negative inputs, respectively.

The first issue addressed in this study is how to obtain an electrochemical output signal from two inputs to use metalloprotein in a hardware-type logical device. To obtain a single output regulated by input information, the electrochemical signal of myoglobin was investigated with various organic spacers (2MAA, 3MPA, 6MHA, 8MOA, 11MUA, and 16MHA). Figure 2a shows that S1 and S2 can be controlled using various organic spacers with two to sixteen carbons. As the organic spacer was lengthened, S1 shifted to the negative direction and S2 decreased due to the delay of electron transfer. This phenomenon is caused by changes in the electron transfer rate between metalloproteins and the metal surface due to the distance variation induced by the organic spacers^16^,^17^. The output signal can be arithmetically calculated when the same information is input (Fig. 2b). This implies that each response from myoglobin on each electrode can be arithmetically operated on without any weighting or interactive factors (independent region represented in Fig. 1c).

In the case of the interactive logic region, the single output signal needs to present the quantity and polarity of information inputs from several electrodes. The second issue addressed was how to obtain a single electrochemical final output by merging independent output signals generated from two different inputs (positive and negative). Figure 2c shows that two independent output signals (positive input P1 from the myoglobin/2MAA electrode and negative input N1 from the myoglobin/3MPA electrode) had merged well into a single output signal (P1N1) when we used the 2MAA/3MPA organic spacer set. The final output signal can be regulated by the ratio of positive and negative information.

Figure S4 shows the final output signal shifted from a positive result (myoglobin/2MAA dominant) to a negative result (myoglobin/6MHA dominant) based on the ratio of the organic spacer when the 2MAA/6MHA set was applied. This result demonstrates the key logic tools considered in this study. The final output logical result can be regulated by the input information (based on the positive and negative information ratio and information polarity). The changing magnitude of the final output signal depends on the organic spacer set. Therefore, the logic device can be demonstrated by combining the metalloprotein/organic spacer set with the metalloprotein acting as a logical center and the organic spacer set defining an already experienced (or educated) tendency (a pre-determined condition).

We defined TP to represent the experienced tendency set with an organic spacer. TP is the ratio of the initial signal intensities (N1/P1; S2 value by first negative input/S2 value by first positive input). As shown in Fig. 2a, the electrochemical signal of myoglobin changed according to the length of the organic spacer. Figure 2d shows a schematic of the interactive logical region, which can be determined by the interaction of positive and negative inputs via the organic spacer set and metalloprotein. This position represents a personal tendency, and the size is related to the decision flexibility.

We changed all output results to a normalized value ranging from 0 to 1 based on the output results of the independent logical region and the two concepts of reliability (absolute negative: −100%; absolute positive: 100%) and confidence (0 to 100%), similar to the representation in Fig. 1a. To demonstrate a human experience-dependent logical result, we devised a balanced device (TP = 0.8, darker regions in Fig. 2d) and a one-sided device (TP = 0.5, brighter regions in Fig. 2d) with the spacer set of 2MAA and 3MPA and the set of 3MPA and 6MHA, respectively.

The interactive logical results of the balanced device are shown in Fig. 2e. This final decision result was influenced similarly by both the negative and positive inputs. When positive and negative information was input (P3N1 is defined as three positive inputs and one negative input), the interactive region in the case of balanced logic was almost at the center between the positive and negative positions. The one-sided logic device is shown in Fig. 2f. As expected, the organic spacer set with the lower TP presented a more one-sided biased personality, in which excessively optimistic tendencies were revealed by the position of the interactive logical region. This device gave a slightly negative decision when only three negative inputs and less than two positive inputs were applied.

Figure 2e,f show that the logical results can significantly differ from human tendencies, even if the same input information is introduced. For example, when 2 positive inputs and 1 negative input (2P1N) were applied to the balanced logic device, the decision result indicated 88% positive and 79% reliable compared with the independent logic result when 3 positive inputs were used. These two simple demonstrations imply that it is possible to apply an “experienced personality” to an electronic device to make an analog decision using an organic spacer set.

### The changes of interactive logical regions

**Figure 3:**
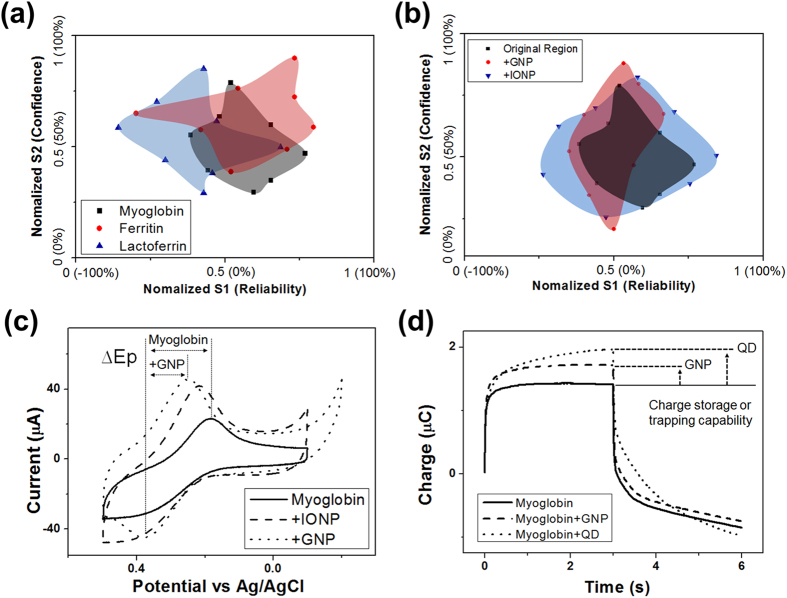
(**a**) Changes of the interactive logical region due to variation of the metalloprotein with the same 2MAA/3MPA organic spacer. (**b**) Changes of interactive logical regions according to the nanoparticles applied. (**c**) Electrochemical signal changes of immobilized myoglobin on 2MAA organic spacer due to nanoparticles. (**d**) Chronocoulometry results confirming variation of the amount of charged electrons by applying GNP and QD.

Figure 3a shows various interactive logical regions generated from different metalloproteins for when the 2MAA/3MPA organic spacer set was chosen. As expected, different metalloproteins provide different interactive logical regions resulting from different logical results. We used this metalloprotein to represent a person’s inherent tendency. The variation in interactive logic results therefore implies that an inherent personal tendency can affect the logic process. When we employed lactoferrin instead of myoglobin, a pessimistic person’s decision logic can be represented. In the case of Ferritin, we can say this device has more confidence than myoglobin because the S2 value was higher.

When we make a decision based on various information inputs, the final decision can be affected by the *in situ* environment and personal inherent and educated tendencies. Therefore, we need to present an environment-dependent logic system. We defined specific ions or nanoparticles that can bind to metalloprotein and an electrolyte solute as environmental factors. When the environment changed (when a nanoparticle was introduced), the interactive logical regions varied in terms of shape and area, as shown in Fig. 3b. The interactive logic region became narrower with nanoparticles compared to the original region (myoglobin without any nanoparticles). When iron oxide nanoparticles (IONPs) were applied, the logic became more balanced.

Sometimes, a person can be considerably shocked by an environment where nothing can be judged. We can present such an environment by simply introducing quantum dots (QDs). The electrochemical signal of the metalloprotein disappeared when a QD was applied (Figure S5). This result implies that the device makes it possible to develop an environment-dependent logic system. Signal changes due to the introduction of nanoparticles can be explained by electron trapping and the reinforcement of electron transfer. The QD is a semiconductor material with a core-shell dual structure. It was assumed that the electrons generated from the redox reaction of metalloprotein were trapped in the QD structure by a mechanism similar to a hysteresis phenomenon due to the difference in band gap^18^,^19^. We reported signal reinforcement using conducting materials such as GNP in previous work^20^. This was based on increasing the conductivity, which resulted in fast electron transfer and quantitative augmentation of the redox-reactive metalloprotein. The theoretical mechanism is shown in supplementary Figure S6.

∆Ep, which is the variation range defined by the difference between Epc and Epa of practical redox peaks, was increased by delay of the reaction, whereas an ideal reversible reaction was operated at the same potential (Epc = Epa). Results that are in accordance with this theory are shown in Fig. 3c. ∆Ep was decreased by the introduction of GNP, implying that the conducting nanoparticles acted as a mediator for fast electron transfer. This phenomenon was also confirmed by chronocoulometry, which is a technique for measuring the amount of charge produced by an applied potential, as shown in Fig. 3d. The absolute charge amount of GNP-modified myoglobin was considerably higher than the result of myoglobin only, although the charging and discharging curves converged with similar shapes. However, the charge amount of QD-modified myoglobin was increased continuously, and the charged electrons were then released more slowly than in the case of GNP. These results indicate that QD can trap generated electrons, in contrast to GNP, which only reinforces the electron transfer capability.

When metal ions were applied to the metalloprotein by electrostatic interaction, the position and intensity of the redox signals were altered because the ions performed redox reactions with changes in ionic valency (Figure S7a). We selected three types of metal ions with a stable signal to confirm the variation of interactive logical regions, and the results are shown in supplementary Figure S7b. The electrochemical signals of immobilized metalloproteins were also affected by different ions with different concentrations in the electrolyte solution, resulting in variation of the interactive logical regions by the same mechanism as that of the conducting nanoparticles (Figure S8).

### Demonstration of group decision-making process

Each value of the investigated interactive logical region can be directly applied to a practical decision process in an optional event by employing a threshold value for the final decision. As shown in Fig. 1a, the decision process in our conceptual demonstration has an ambiguous region that has not yet been determined. However, “positive” and “negative” polarities appear, in contrast to digitalized logic with only two outputs of “yes” and “no.” This approach provides benefits in describing relativeness and ambiguity in an intricate logic process under ambiguous conditions. The proposed logic device can represent various logic phenotypes governed by inherent and experienced tendencies and environmental stimuli.

**Figure 4:**
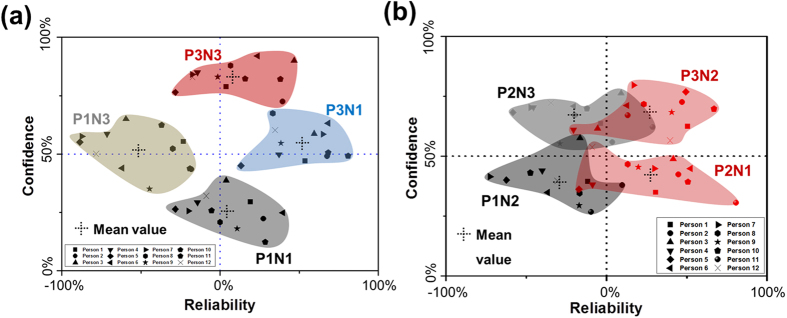
(**a**) Decision variation of 12 people with a clear tendency for the input combination of P1N1, P1N3, P3N1, and P3N3. (**b**) Decision results with ambiguous tendency for the input combination of P1N2, P2N1, P2N3, and P3N2.

As a practical application, this artificial biologic device can be directly applied to mimic a group decision. Figure 4 shows the individual decisions in an imaginary group of 12 people who have different tendencies in different environments. The people’s tendencies were defined using the mean value and variation range of individual interactive logical regions (Figure S9). Six items of information were input in total. This imaginary group is normally balanced, but slightly optimistic characteristics were observed due to the TP value (0.8) of the 2MAA/3MPA organic spacer set (refer to the mean values of P1N1 and P3N3 in Fig. 4a).

When one negative and one positive information source (P1N1, 33% input) were input, 6 people thought it was positive and 5 people thought it was negative. However, nobody was able to make a decision. When more negative information (P1N3) or positive (P3N1) information was input, the people provided all negative thoughts or all positive thoughts with increasing confidence. When all the information was input with the same ratio (3:3), every person had enough confidence to make a decision. However, the decision appeared to be one with a P1N1 input with different confidence levels (Fig. 4a).

In the case of an ambiguous information set (P2N1 and P1N2 in Fig. 4b), the group decision became unclear. As shown in Fig. 4b, not all people followed the information ratio. Most people did not have sufficient confidence to make a decision. When more information was input and reached 83% (5 information inputs out of a total of 6), all people had sufficient confidence to make a decision. However, not all people made the same decision. If this group decided to achieve a final decision by voting, it would have more positive decisions for P3N2 with about 83% “yes” and 17% “no”. As a result, we believe this interactive logic device can predict group decisions when an individual person’s tendency is represented by the device. A simple practical example for decision prediction is described in supplementary Figure S10. The dots were fitted by mean values of each input combination, in which the diamond-shaped region shown by dotted lines for equations was well formed. This meant that we can predict some other results with limited data by using linear equations.

## Discussion

The electrochemical signal of metalloprotein was well manipulated by applying organic and inorganic materials. Several functions including enhancement, suppression, and shift were demonstrated by the formation of bio-hybrid materials with various constitutions of organic and inorganic materials. Based on the proposed functions, we demonstrated a human-mimicking bio-logic device that provides analog logical results affected by device characteristics (inherent and experienced personal characteristics) and environments. The inherent device characteristics were demonstrated by metalloprotein, and an organic spacer set was used to represent an experience-induced tendency. An environmental effect was also demonstrated using inorganic materials.

Many challenges are involved in attempts to materialize a human-like logic system, such as a practical logic algorithm or artificial recognition system, because human logic processes are still too intricate to be understood. Therefore, we focused on the apparent characteristics of the human logic process. We believe that the decision flexibility of the logical device, which is affected by tendencies and the environment, resembles a human logic system by providing a final decision result by the combination of memories (experience) and situation (environment) based on personal tendencies. With this result, we can express the effect of ambiguity of input or the combination effect of input/environment/personal tendencies with the proposed device. The logical concept proposed in this research can be used for most systems where there are interferents that are generally ignored while presenting data. The concept can be applied to systems where selectivity towards more than one variable is present. Finally, we believe this demonstration can facilitate the realization of an artificial humanoid-logic intelligent system that interfaces directly with a human brain, even though this can only partially demonstrate similarity to analog human logic.

## Methods

### Materials

Myoglobin extracted from horse heart (where a metalloprotein is used to transfer oxygen using the ionic state change of iron ions) was purchased from Sigma-Aldrich (St. Louis, MO., USA). 2-mercapto acetic acid, 3-mercaptopropionic acid, 6-mercaptohexanoic acid, 8-mercaptooctanoic acid, 11-mercaptoundecanoic acid, 16-mercaptohexadecanoic acid, cysteamine hydrochloride, 1-ethyl-3-(3-dimethylaminopropyl) carbodiimide (EDC), N-hydroxysuccinimide (NHS), and rhodamine B were also purchased from Sigma-Aldrich. The developer Su-8 2 and the remover PG were purchased from Microchem (USA). 10 mM 4-(2-hydroxyethyl)-1-piperazineethanesulfonic acid (HEPES) with pH 7.0 adjusted by sodium hydroxide (NaOH) and hydrochloric acid (HCl) was used for the preparation of protein solution. Distilled and deionized (DI) water with a resistance of less than 18 MΩ produced by a Mili-Q system was used in all experiments to prepare the chemical solution and to rinse the substrate.

### Device design and fabrication

As shown in Figure S1, multi-electrodes were designed with 4-μm gaps between the counter electrode and the six individual working electrodes (resistances R_G1, G2, G3, G4, G5, G6_). The total resistance of each working electrode was calculated to be identical to all other electrodes by two separate resistance calculations based on the region (in contact with the electrolyte and air) for 1) internal regions (indicated by a red dotted line) in contact with electrolyte solution inside the working chamber (R_S1, S2, S3, S4, S5, S6_) and 2) an external region in air (R_L1, L2, L3, L4, L5, L6_). If identical materials are deposited with the same thickness, the resistance can be determined as a function of the length (R = f (L)) based on the equation R = ρL/A. Therefore, all electrode patterns were designed with the same length.

The redox property of biomaterials immobilized on the electrode is represented using the capacitance (C_1, 2, 3, 4, 5, 6_) as an independent characteristic of the electrode, because the electrical properties of all working electrodes were controlled to have the same values. Each electrode was connected with the main working electrode by separate switching. Silicon substrate (100) covered with SiO_2_ produced by thermal oxidation was initially cleaned with piranha solution (H_2_SO_4_ and H_2_O_2_, 5:1 (v/v)) at 70 °C for 10 min and baked at 200 °C for 20 min. The multi-electrodes were patterned using a conventional lift-off process. Su-8 2 photoresist was used as a negative photoresist (3000 rpm). Au (50 nm) was deposited on a Cr layer (5 nm), which was used as an adhesion layer. A lift-off procedure was performed using PG remover for 2 hr at 70 °C. The cleaning procedure with piranha solution was carried out after the lift-off process. The working chamber (400 μl, PDMS) was placed on the multi-electrodes.

### Selective immobilization

Organic spacers (1 mM) dissolved in ethanol were initially immobilized on a multi-electrode for 3 hr at room temperature. A reductive cleavage of the thiol group in the organic spacers was used for the selective immobilization process. The covalent bonding between the gold surface and sulfur at the end of the spacers was easily broken by reduction using an electrochemical method^21^,^22^,^23^, although the binding energy was relatively high at 168 kJ/mol^24^. A schematic of the process is shown in Figure S2.

Firstly, a multi-electrode composed of six individual electrodes was initially modified with one species of mercapto-acid using a self-assembly process. The immobilized organic spacers on one electrode selected from six electrodes were detached from the electrodes by the negative potential for immobilization of another material. After reductive cleavage of the pre-immobilized spacer, a re-immobilization process was performed by immersion into another species of mercapto-acid solution. These steps were performed repeatedly until the composition of the spacers on each electrode was completed. Finally, myoglobin (0.2 mg/ml in 10 mM HEPES buffer of pH 7.0) was deposited on all electrodes using the electrostatic interaction between the carboxyl group of organic spacers and the amine group of proteins for 3 hr at 5 °C. All experiments using metalloprotein were performed immediately after fabrication of the device, and devices were maintained at 5 °C in the case of short-term storage to prevent inactivation and contamination.

The potential for reductive cleavage was generated by a potentiostat (CHI-660, CH Instruments, Inc., TX, USA). The redox properties of immobilized materials on the gold surface were also confirmed by cyclic voltammetry (CV). Parallel-patterned gold electrodes obtained by photolithography, Pt wire, and Ag/AgCl in saturated NaCl (3 M) were used for the working, counter, and reference electrodes, respectively. All electrochemical measurements on the multi-electrodes were performed in a 10 mM HEPES buffer at pH 7.0 and a scan rate of 50 mV/s. The potential range of the working chamber was from 0.5 to −0.2 V, and the volume was 300 μl.

All cyclic voltammograms shown in this research were obtained after the stabilization process of a redox reaction of over 30 cycles. Selective immobilization was also confirmed by fluorescence microscopy (DMLB 100 s, Leica Microsystems, Germany). Initially, a substrate cleaned with piranha solution was immersed in a 1 mM cysteamine solution dissolved in DI water for 3 hr. Rhodamine B (10 mM) was then attached to the cysteamine layer using EDC/NHS coupling for 2 hr. The experimental results of the selective immobilization process are presented in Figure S3.

### Preparation of inorganic materials for environment factor

The buffer solution of GNP with diameters of 10 nm and 30 nm was initially changed from PBS to methanol for the separation process using a centrifuge. 300 μl of 2MAA was added to 1 mL of GNP solution and reacted for 12 hr. This mixture was washed with pure methanol more than three times, and the 2MAA-modified GNP was then dissolved in HEPES buffer for the deposition process on metalloprotein. 1 mL QD in chloroform precipitated from toluene with methanol was reacted with 300 μl of 2MAA for 24 hr. This mixture was washed with methanol more than three times and finally dissolved in HEPES buffer.

Iron oxide nanoparticles were initially dissolved in DI water at pH 11 and reacted with APTES ((3-Aminopropyl)triethoxysilane) for more than 6 hr. More than 3 washing steps were performed with DI water and a magnet. After surface modification, all nanoparticles were sonicated to make a well-dispersed solution. The prepared nanoparticles were immobilized on the myoglobin modified with organic spacing materials for 3 hr at 5 °C by electrostatic interaction between opposite charges. Nickel(II) chloride, manganese(ii) sulfate, copper(ii) sulfate, iron(ii) chloride, and cobalt(ii) chloride were initially dissolved in HEPES buffer at a concentration of 50 mM. Metal ions were then reacted with immobilized metalloprotein for 3 hr at 5 °C, followed by sufficient rinsing with DI water.

## Additional Information

**How to cite this article**: Chung, Y.-H. *et al.* Electrochemical Bioelectronic Device Consisting of Metalloprotein for Analog Decision Making. *Sci. Rep.* **5**, 14501; doi: 10.1038/srep14501 (2015).

## Supplementary Material

Supplementary Information
