# Supplementary material for: Electrochemical Bioelectronic Device Consisting of Metalloprotein for Analog Decision Making
Source: Sci Rep. 2015 Sep 24;5:14501. doi: 10.1038/srep14501 (PMC4585857; doi:10.1038/srep14501)
Supplement: Supplementary Information [file srep14501-s1.pdf]

Supplementary information

Electrochemical Bioelectronic Device Consisting of Metalloprotein for Analog Decision Making

Yong-Ho Chung, Si-Youl Yoo, Taek Lee, Junhong Min*, and Jeong-Woo Choi*

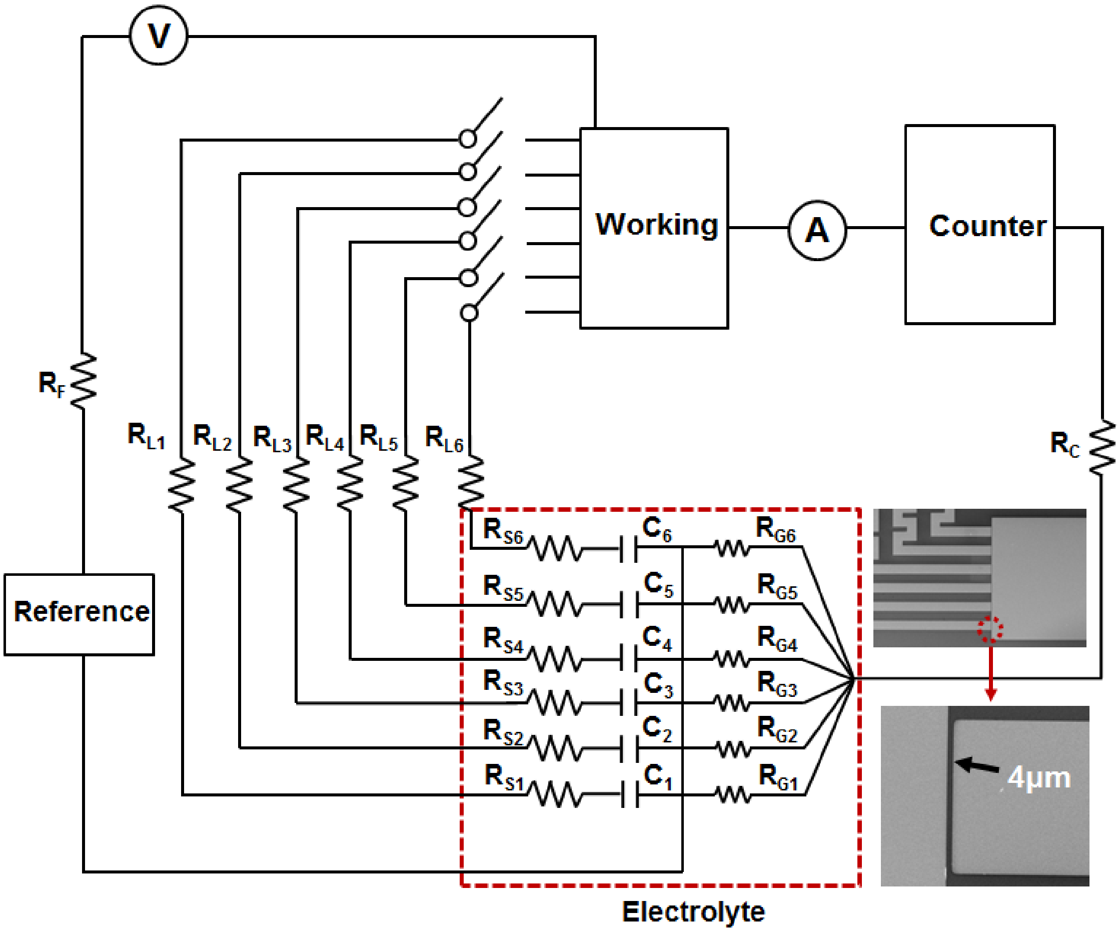

Figure S1. Bio-electronic device design: circuit diagram of electrochemical detection system using multi-electrode (R: resistance, C: capacitance)

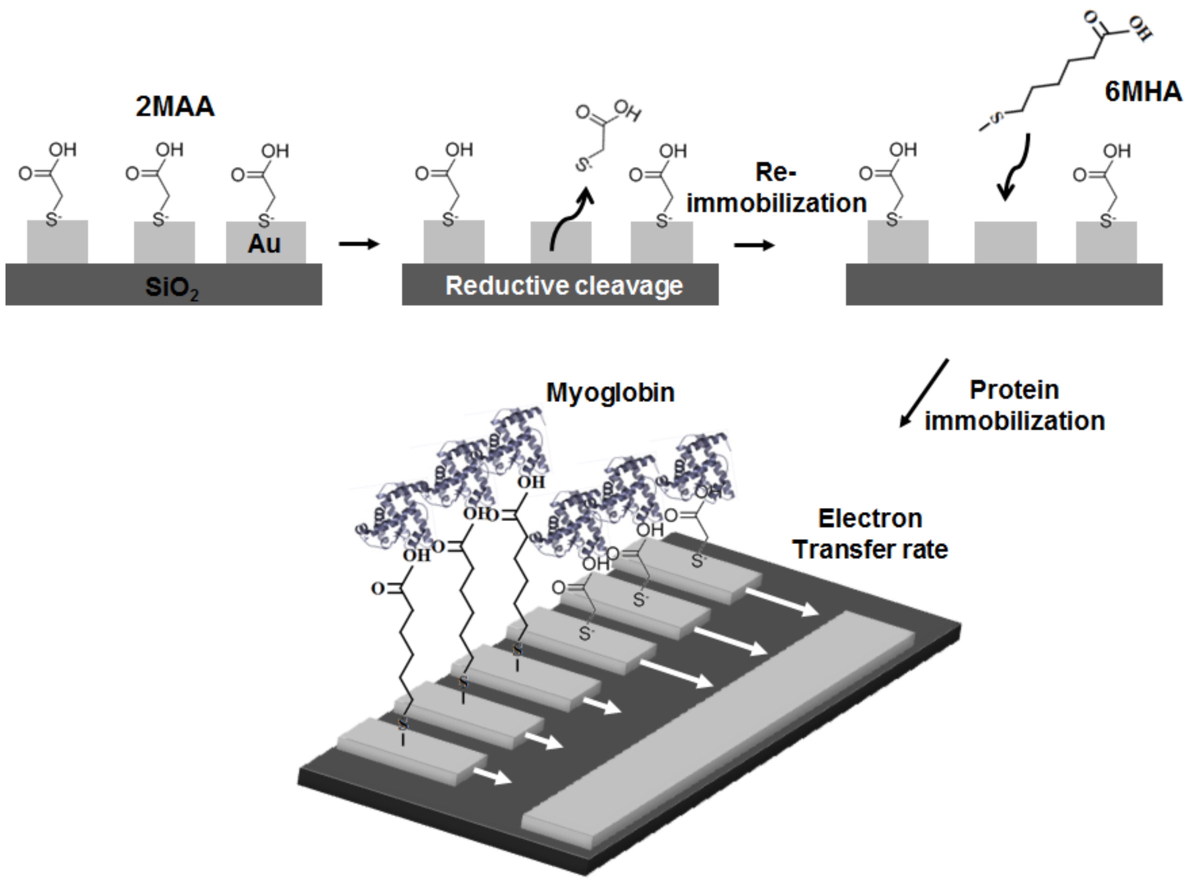

Figure S2. Selective immobilization process: sequential schematic diagram of selective immobilization process using reductive cleavage of thiol-gold bonding.

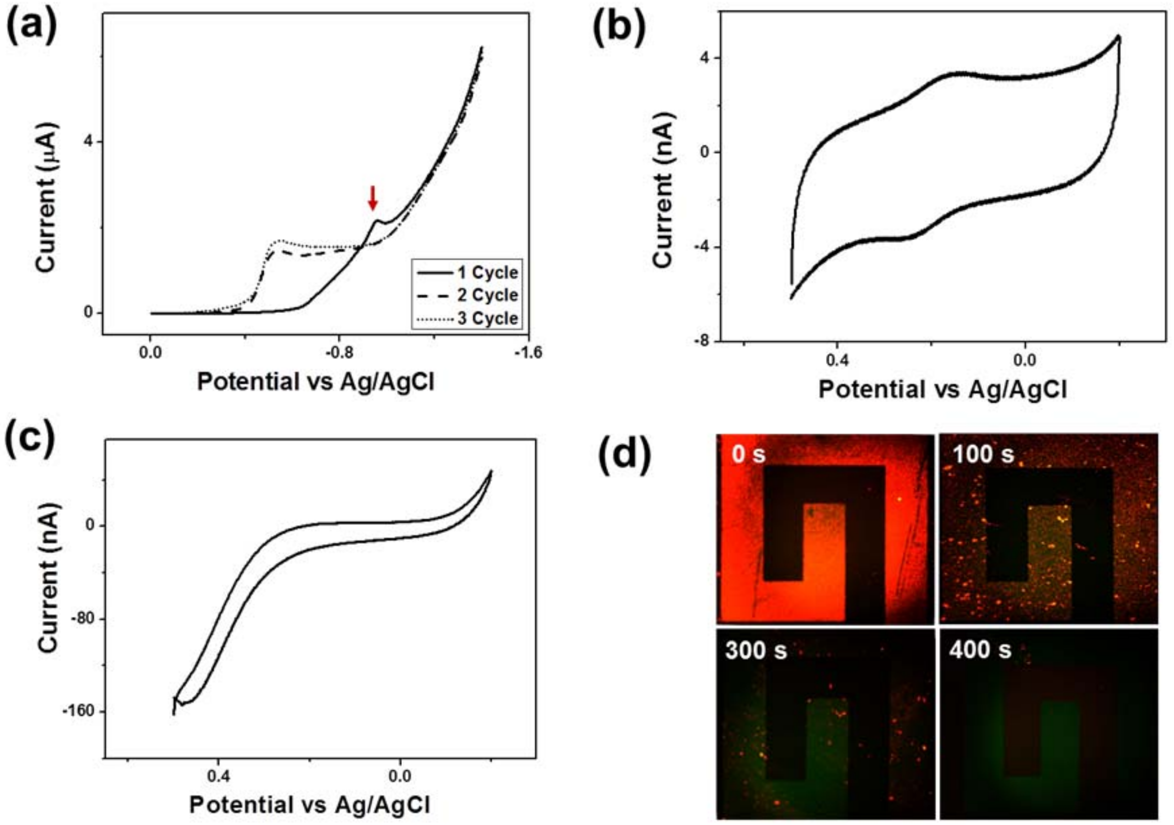

Figure S3. (a) I-V graph according to applying negative potential continuously with the form of cycle for reductive cleavage of covalent bonding between gold and sulfur, (b) Cyclic voltammogram of 2MAA/Myoglobin immobilized on gold surface before reductive cleavage, (c) Cyclic voltammogram after applying negative potential of -1.4 V for 100 s, (d) Changes of fluorescence intensity by reductive cleavage of rhodamine B with the time variation.

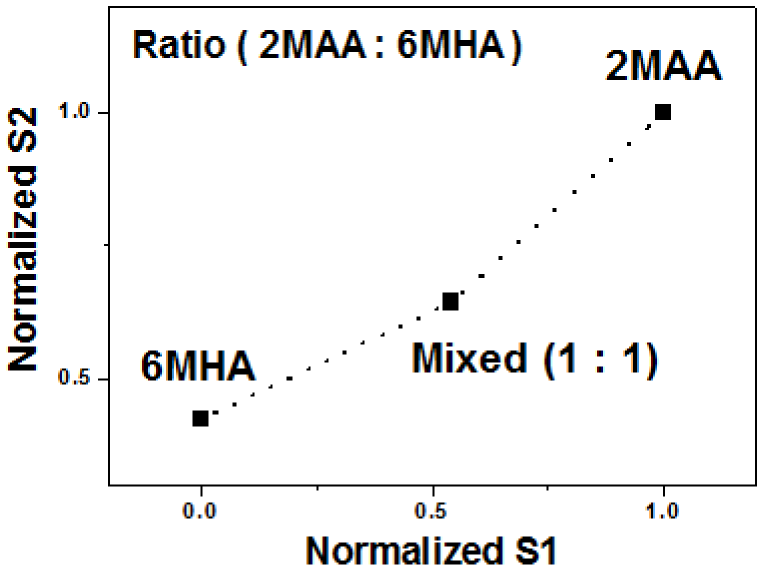

Figure S4. Signal changes of immobilized myoglobin on the two different spacers according to the ratio (2MAA: 6MHA, initial immobilized materials: 2MAA, re-immobilized material: 6MHA).

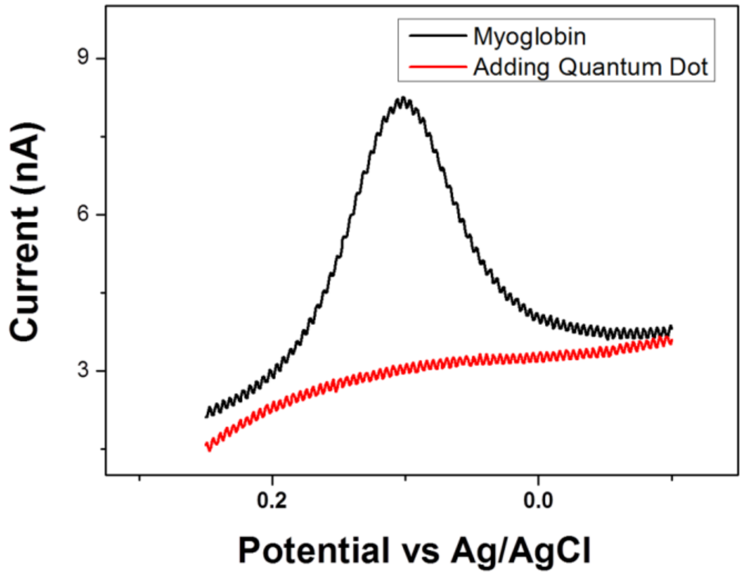

Figure S5. Signal change due to the introduction of quantum dot.

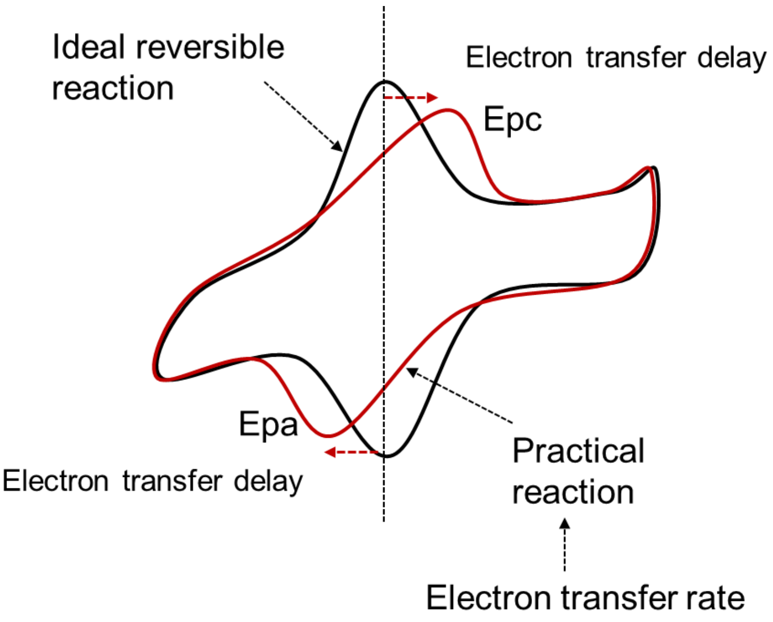

Figure S6. Theoretical phenomenon related with peak shift by electron transfer delay, compared with ideal reversible reaction.

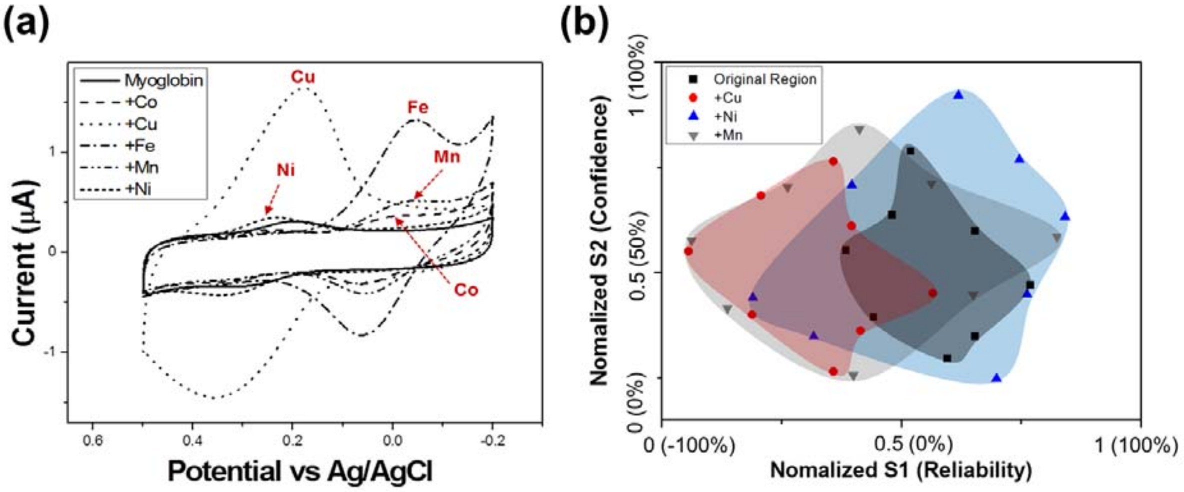

Figure S7. (a) Cyclic voltammogram with various metal ions using bulk-patterned electrode as the comparison with the signal of myoglobin only. (b) Variation of interactive logical regions using micro-patterned electrode according to the combination of metal ions.

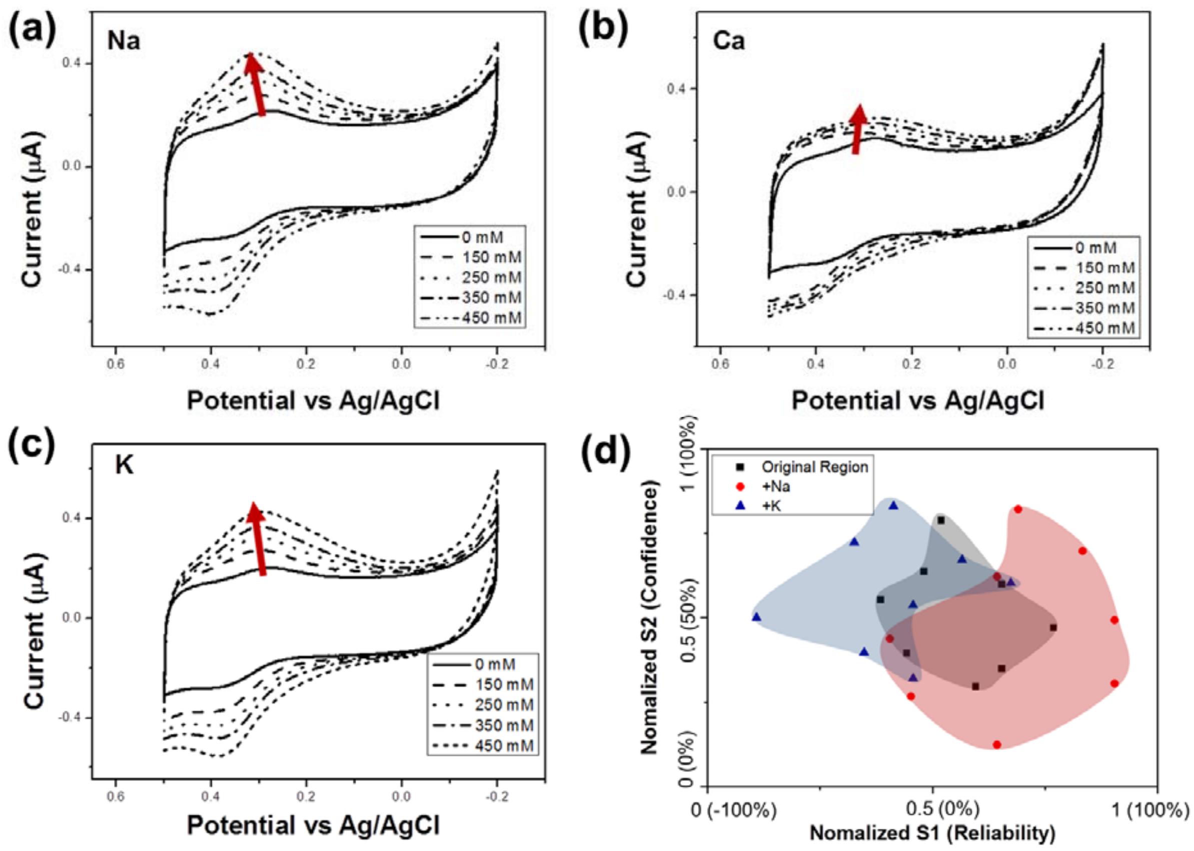

Figure S8. (a) Cyclic voltammogram according to the concentration change of sodium ions on bulk-patterned electrode. (b) Calcium. (c) Potassium. (d) The variation of interactive logical regions by applying electrolyte ions in solution on micro-patterned electrode.

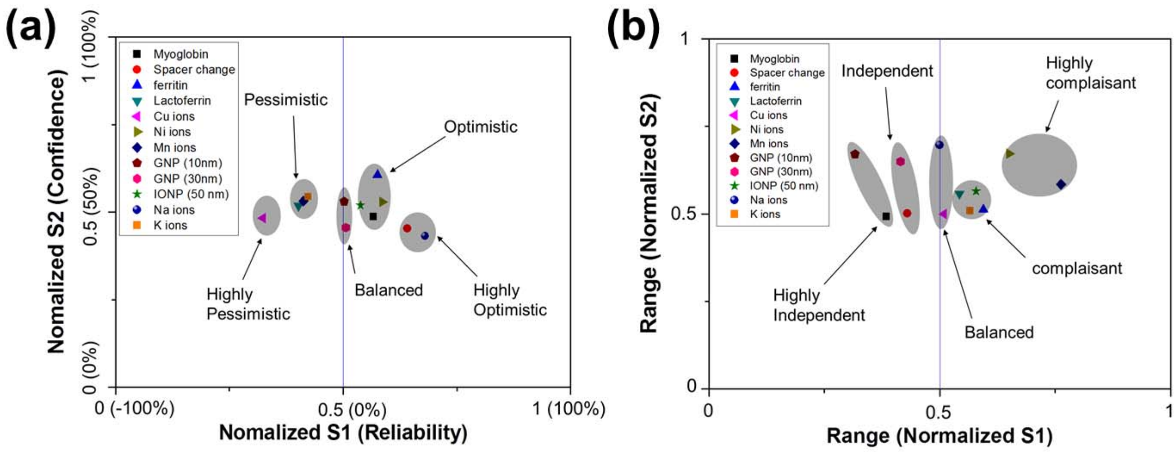

(c)

	Inherent tendency	Educated tendency	Environmental factor	Defined characteristics	
				Basic tendency	Subjective tendency
Person 1	Myoglobin	2MAA/3MPA	None	Optimistic	Highly independent
Person 2	Myoglobin	**3MPA/6MHA**	None	Highly Optimistic	Independent
Person 3	**Ferritin**	2MAA/3MPA	None	Optimistic	Complaisant
Person 4	**Lactoferrin**	2MAA/3MPA	None	Pessimistic	Complaisant
Person 5	Myoglobin	2MAA/3MPA	**Cu ions**	Highly pessimistic	Balanced
Person 6	Myoglobin	2MAA/3MPA	**Ni ions**	Optimistic	Highly complaisant
Person 7	Myoglobin	2MAA/3MPA	**Mn ions**	Pessimistic	Highly complaisant
Person 8	Myoglobin	2MAA/3MPA	**GNP (10 nm)**	Balanced	Highly independent
Person 9	Myoglobin	2MAA/3MPA	**GNP (30 nm)**	Balanced	Independent
Person 10	Myoglobin	2MAA/3MPA	**IONP (50 nm)**	Optimistic	Complaisant
Person 11	Myoglobin	2MAA/3MPA	**Electrolyte ions (Na+)**	Highly optimistic	Balanced
Person 12	Myoglobin	2MAA/3MPA	**Electrolyte ions (K+)**	Pessimistic	Complaisant

Figure S9. (a) Definition of basic tendency using mean value of individual interactive logical results, (b) Definition of subjective tendency using range of S2 value, (c) Defined characteristics of 12 persons

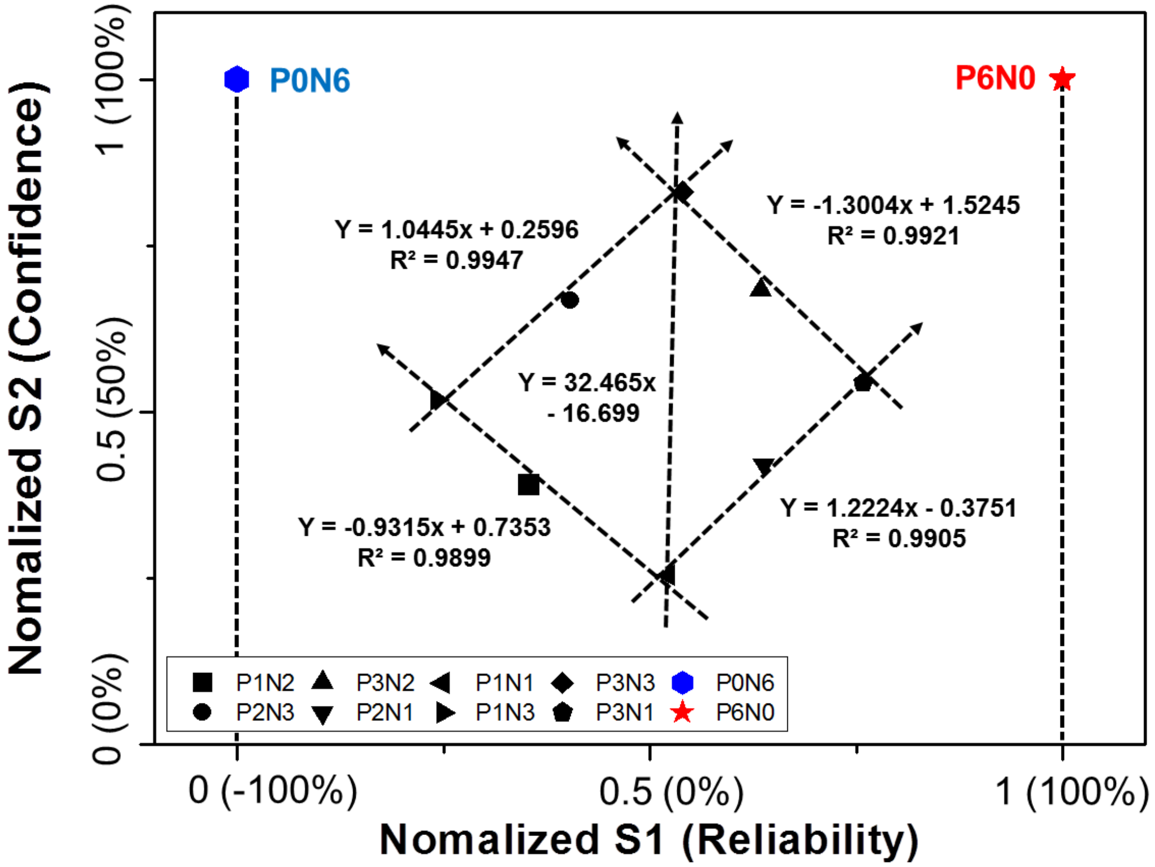

Figure S10. Prediction model for group decision by linear equation. The represented all points were fitted by mean values. And dotted lines meant calculated linear equations.
